# Platelet‐inspired nanomedicine in hemostasis thrombosis and thromboinflammation

**DOI:** 10.1111/jth.15734

**Published:** 2022-04-26

**Authors:** Shruti Raghunathan, Julie Rayes, Anirban Sen Gupta

**Affiliations:** ^1^ 2546 Department of Biomedical Engineering Case Western Reserve University Cleveland Ohio USA; ^2^ Institute of Cardiovascular Sciences College of Medical and Dental Sciences University of Birmingham Birmingham UK

**Keywords:** haemostasis, nanomedicine, platelets, thromboinflammation, thrombosis

## Abstract

Platelets are anucleate cell‐fragments derived predominantly from megakaryocytes in the bone marrow and released in the blood circulation, with a normal count of 150 000–40 000 per μl and a lifespan of approximately 10 days in humans. A primary role of platelets is to aid in vascular injury site‐specific clot formation to stanch bleeding, termed hemostasis. Platelets render hemostasis by a complex concert of mechanisms involving platelet adhesion, activation and aggregation, coagulation amplification, and clot retraction. Additionally, platelet secretome can influence coagulation kinetics and clot morphology. Therefore, platelet defects and dysfunctions result in bleeding complications. Current treatment for such complications involve prophylactic or emergency transfusion of platelets. However, platelet transfusion logistics constantly suffer from limited donor availability, challenges in portability and storage, high bacterial contamination risks, and very short shelf life (~5 days). To address these issues, an exciting area of research is focusing on the development of microparticle‐ and nanoparticle‐based *platelet surrogate* technologies that can mimic various hemostatic mechanisms of platelets. On the other hand, aberrant occurrence of the platelet mechanisms lead to the pathological manifestation of thrombosis and thromboinflammation. The treatments for this are focused on inhibiting the mechanisms or resolving the formed clots. Here, platelet‐inspired technologies can provide unique platforms for disease‐targeted drug delivery to achieve high therapeutic efficacy while avoiding systemic side‐effects. This review will provide brief mechanistic insight into the role of platelets in hemostasis, thrombosis and thromboinflammation, and present the current state‐of‐art in the design of platelet‐inspired nanomedicine for applications in these areas.

## 1 INTRODUCTION

Platelets are anucleated cells released from membrane protrusions (proplatelets) of mature megakaryocytes, and circulate in the human blood at a healthy count of 150 000–40 000 per μL, with a lifespan of approximately 10 days.^1^ A primary role of platelets is in forming hemostatic clots to stop bleeding. Therefore, defects in platelet number and functions lead to bleeding complications.^2^ Current treatment for such complications involve prophylactic or emergency transfusion of platelets. However, platelet transfusion logistics suffer from limited donor availability, challenges in portability and storage, high bacterial contamination risks, and very short shelf life (~5 days). To address these issues, an exciting area of research is focusing on the development of nanoparticle‐based *platelet surrogate* technologies that can mimic hemostatic mechanisms of platelets. The same mechanisms by which platelets aid in hemostatic clot formation, if dysregulated, can result in unwanted clots (thrombosis) e.g. in heart attack, stroke, etc.^3^, ^4^. Furthermore, heterotypic interactions between platelets, vascular endothelium and leukocytes have been implicated in thromboinflammation, a pathological phenotype implicated in deep vein thrombosis, sepsis, trauma and emerging COVID‐19 pathology.^5^, ^6^ Therefore, significant therapeutic development has focused on elucidating and modulating these roles of platelets. Here, platelet‐inspired nanotechnologies can provide unique platforms for disease‐targeted drug delivery to achieve high therapeutic efficacy while avoiding systemic side‐effects. In this review, we aim to provide brief mechanistic insight into the role of platelets in hemostasis, thrombosis and thromboinflammation, and present the current state‐of‐art along and future opportunities, in the design and application of platelet‐inspired nanomedicine in these areas.

## 2 PLATELET MECHANISMS IN HEMOSTASIS AND AND PLATELET TRANSFUSION IN BLEEDING MANAGEMENT

**FIGURE 1:**
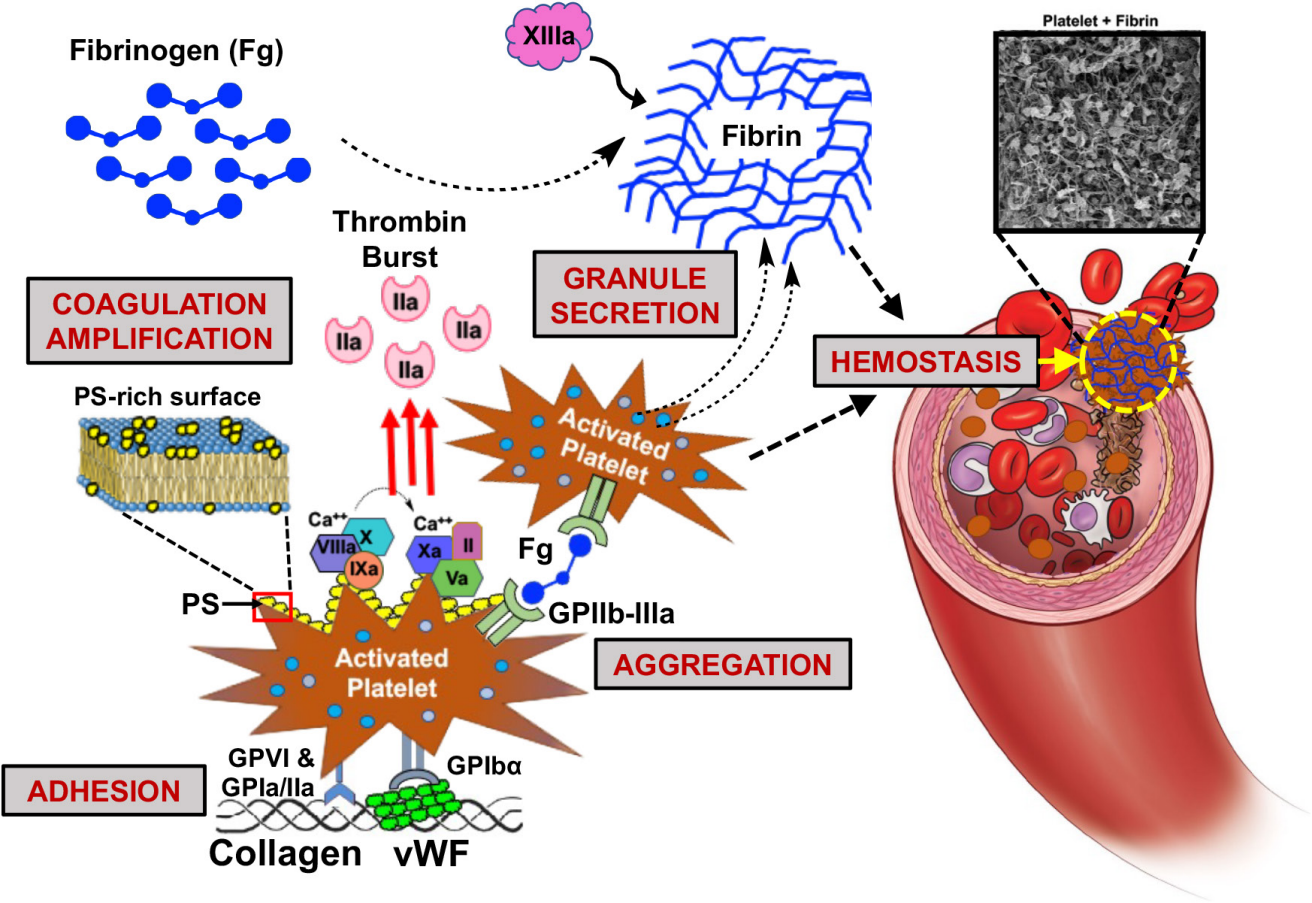
Platelet mechanisms in hemostasis involving platelet adhesion to von Willebrand Factor (vWF) and collgen, aggregation mediated by fibrinogen (Fg), coagulation amplification via surface presentation of phosphatidylserine (PS) to render the ‘thrombin burst’ for localized generation of fibrin from fibronogen, and secretion of granule contents to augment coagulation kinetics and fibrin morphology; Platelets and fibrin form the hemostatic clot to stop bleeding

Platelets render hemostasis by a complex concert of mechanisms (Figure 1): (1) Rapid *adhesion* under flow to injury site‐exposed von Willebrand Factor (vWF) and collagen; (2) Fibrinogen (Fg)‐mediated *aggregation* of activated platelets at the injury site; (3) Presentation of anionic phospholipid‐rich procoagulant platelet surface to facilitate *thrombin amplification*; (4) *Secretion* of several clot‐promoting molecules from cytoplasmic granules (e.g. vWF, Adenosine diphosphate or ADP, inorganic polyphosphate or PolyP, etc.) and membrane lipid processes (e.g. thromboxane A2 or TXA_2_) to augment clot kinetics and morphology; and (5) Facilitating *clot retraction* by inducing contractile forces via platelet surface integrin GPIIb‐IIIa binding to and pulling on fibrin.^7^, ^8^, ^9^, ^10^, ^11^, ^12^, ^13^, ^14^, ^15^ Consequently, defects in platelet number and functions, can lead to bleeding risks and hemorrhage. This is evidenced in platelet defects like Immune Thrombocytopenia (antibody‐induced platelet clearance leading to a ‘count’ defect), Glanzmann Thrombasthenia (genetic defect in platelet GPIIb‐IIIa causing impaired platelet aggregation), Bernard‐Soulier Syndrome (genetic defect in platelet GPIb‐IX‐V causing impaired platelet adhesion), and surgery‐ or trauma‐induced platelet depletion and dysfunction. Platelet transfusions are often necessary to reduce bleeding risks or mitigate hemorrhage in such scenarios.^16^, ^17^, ^18^, ^19^, ^20^ However, platelet transfusion products are dependent on donor blood availability, which remains a persistent global challenge.^21^ Additionally, donor‐derived room temperature stored platelets (RT‐Plt) have a high risk of bacterial contamination as well as activation/degranulation upon storage, and their shelf‐life is only 5–7 days.^22^, ^23^

Significant strategies are currently focused on reducing contamination risks and increasing platelet shelf‐life by pathogen reduction technologies, storing platelets at low temperatures (e.g. cold‐stored and cryopreserved), and processing platelets at reduced temperature (e.g. freeze‐drying).^24^, ^25^ Cooling or lyophilizing platelets have been around since the 1950s and cryopreservation since the 1970s, but their widespread use in platelet transfusion products has not been adopted broadly yet. This is partly due to the rapid clearance of cold‐stored and lyophilized platelets from circulation by hepatic macrophages (circulation lifespan ~1.5 days compared to ~4 days for RT‐Plt).^26^, ^27^, ^28^, ^29^, ^30^ Reduced temperature processing and storage also induce functional changes in platelets, including desialylation, GPIbα clustering, partial loss of GPIIb‐IIIa function, increased activation, procoagulant phosphatidylserine (PS) exposure and formation of a higher percentage of microparticles (collectively termed ‘cold storage lesion’), which accelerate their clearance and limit their therapeutic potential.^31^, ^32^ However, some of these functional changes (e.g. increased PS exposure) may make these platelets *hemostatically primed* for rapid clot formation and therefore they are currently being studied for emergency management of active hemorrhage.

## 3 PLATELET‐INSPIRED HEMOSTATIC NANOMEDICINE

### 3.1 Nanotechnologies utilizing platelet‐derived membrane components

While several approaches described above are partly improving the transfusion logistics of donor‐derived platelets, parallel scientific endeavors are exploring whether platelet's hemostatic mechanisms can be simulated on biosynthetic nanoparticle systems. In such platelet‐inspired *reductionist design*, specific platelet mechanisms are mimicked by biomolecular surface‐modification of particle platforms. The earliest approaches in this area utilized detergent‐mediated extraction of platelet membrane glycoproteins for incorporation within the membrane of liposomal vesicles, resulting in a design termed ‘Plateletsome’.^33^ The rationale here was that the extracted membrane glycoproteins would retain some hemostatic functions. An evolved variation of this approach led to the product *infusible platelet membrane* (IPM Cyplex, Cypress Bioscience), which utilized extracted and pasteurized membrane from donor‐derived platelets.^34^ IPM vesicles demonstrated promising hemostatic ability in thrombocytopenic rabbit models, and progressed to early phase clinical trials in thrombocytopenic patients. However, further trials were not done, possibly due to complicated manufacturing and scale‐up logistics of IPM, as well as its limited efficacy. It is important to note here that the ‘extracted platelet membrane’ approaches are still dependent on donor platelets.

### 3.2 Nanotechnologies inspired by platelet aggregation mechanisms

Instead of utilizing extracted platelet membrane, some designs have focused on using specific hemostasis‐relevant proteins to coat particles. One such protein is fibrinogen (Fg), which is essential for platelet aggregation via its binding to *activated* platelet integrin GPIIb‐IIIa. Therefore, approaches have focused on coating Fg on RBCs, as well as on albumin‐based synthetic microparticles (e.g. Synthocytes™, Thrombospheres™, Fibrinoplate™ etc.) to create ‘super‐fibrinogen’ constructs that augment the platelet aggregatory kinetics.^35^, ^36^, ^37^, ^38^, ^39^ These Fg‐coated particles have shown promising hemostatic ability *in vivo*, but have not been rigorously evaluated clinically. Of note, human Fg concentrate (e.g. Riastap from CSL Behring) is clinically approved for treating bleeding related to fibrinogen deficiency. Therefore, one can envision that Fg‐coated particles may have similar translational feasibility. Elucidation of the GPIIb‐IIIa‐binding specific domains of Fg has also led to the exploration of using such domain‐relevant peptide sequences to coat micro/nano‐particles. Integrin GPIIb‐IIIa on stimulated platelets binds to Arg‐Gly‐Asp (RGD) and His‐His‐Leu‐Gly‐Gly‐Ala‐Lys‐Gln‐Ala‐Gly‐Asp‐Val (HHLGGAKQAGDV, also known as H12) peptide sequences in the α and γ chains of Fg.^40^ Thus, one of the earliest approaches involved surface‐decoration of RBCs with RGD peptides, resulting in ‘Thromboerythrocyte’ technology.^41^ These constructs could increase the overall aggregation of ADP‐activated platelets. In recent years, this approach has been adapted by using RGD‐peptide motifs to decorate poly‐lactic acid/poly‐glycolic acid (PLA, PLGA) nanoparticles.^42^, ^43^ The RGD sequences used in these designs are CGRGD or GRGDS, that have binding ability to platelet GPIIb‐IIIa, but present two potential limitations: (i) these RGD motifs are highly ubiquitous and bind many different integrins on other cells, and thus lack *platelet‐specificity*, and (ii) they can trigger partial activation of resting platelets, thus posing systemic pro‐thrombotic risks.^44^, ^45^, ^46^ In comparison, the H12 peptide is deemed to have higher specificity to activated platelet GPIIb‐IIIa. Several studies have explored coating this peptide on liposomes, latex beads, and albumin particles to enhance platelet aggregation.^47^, ^48^ These constructs have all shown promising hemostatic effect in preclinical animal models. In a recent approach, H‐12‐decorated liposomes were further loaded with ADP (a platelet agonist) to enhance hemostatic efficacy in rabbit models of thrombocytopenia and hemorrhage.^49^ In our research on mimicking fibrinogen interaction with GPIIb‐IIIa, we have decorated liposomes with a linear RGD (l‐RGD) peptide GSSSGRGDSPA, as well as a cyclic RGD (c‐RGD) peptide cyclo‐CNPRGDY(*OEt*)RC, to demonstrate that the c‐RGD‐decorated liposomes have higher affinity and specificity to *activated* platelet GPIIb‐IIIa, *in vitro* and *in vivo*.^50^ Consequently, we have used liposomes decorated with this c‐RGD peptide (subsequently termed fibrinogen‐mimetic peptide or FMP) to enhance platelet aggregation.^51^ The FMP‐decorated liposomes were able to reduce bleeding time in a tail‐clip injury in mice.^52^ These studies provide evidence that in designing nanoparticles that mimic Fg‐mediated platelet aggregation, it is important to select peptides that have high platelet specificity and affinity. Furthermore, in translational advancement of such technologies, the peptide decoration density as well as total nanoparticle dose will need to be optimized, such that the particles enhance rather than competitively inhibit endogenous Fg‐mediated platelet aggregation. Besides their interaction with Fg monomers, platelet GPIIb‐IIIa also interacts with fibrin at the clot site to render biomechanical contractile forces that govern clot stability.^15^ Inspired by this, a unique design has explored the decoration of poly‐N‐isopropyl acrylamide (Poly‐NIPAM) based low‐crosslinked microgel particles with antibody fragments that bind to fibrin.^53^ These flexible fibrin‐binding particles could mimic platelet‐mediated clot contraction, with the caveat that their binding would require prior presence of sufficient fibrin (i.e. significant coagulation) at the injury site. Therefore, hematologic dysfunctions that present sub‐optimal thrombin generation and fibrin formation (e.g. hemophilia, trauma‐induced coagulopathy, etc.) may require additional refinement of this technology for sufficient hemostatic effect. However, these particles can act as an effective drug‐carrying platform to treat thrombotic diseases, as described in Section 4 later.

### 3.3 Nanotechnologies inspired by platelet adhesion mechanisms

Several design approaches have also explored mimicking the vWF‐ and collagen‐interactive *adhesion* mechanisms of platelets. vWF is secreted from injured endothelial cells as a globular protein, and under shear flow it unravels to expose specific domains with hemostatically relevant bioactivity. Specifically, the A1 domain mediates binding to platelet GPIbα.^54^ This binding is shear‐dependent and reversible, and leads to initial platelet attachment and rolling. The vascular injury site also presents sub‐endothelial collagen as a major matrix component, and platelet surface glycoproteins GPIa/IIa and GPVI bind to collagen. These synergistic vWF‐ and collagen‐binding interactions are critical for rapid platelet adhesion in hemostasis.^7^ Based on this, some approaches have utilized decoration of liposomes, latex beads and albumin‐based microparticles with recombinant GPIbα (rGPIbα) and recombinant GPIa/IIa (rGPIa/IIa).^55^, ^56^, ^57^ These particles could effectively adhere to vWF‐coated and collagen‐coated surfaces *in vitro* under flow. In an additional approach, the rGPIbα and rGPIa/IIa motifs were co‐decorated on liposomes and albumin particles, closely mimicking platelet adhesion mechanisms.^56^ While these are exciting platelet‐inspired approaches, there may be potential translational challenges associated with the high cost of recombinant technology, as well as mutual steric interference between the large recombinant protein fragments co‐decorated on a particle surface. Therefore, subsequent approaches have explored utilization of peptides instead of proteins for particle surface‐decoration to mimic platelet adhesion. To this end, researchers have identified peptides that mediate the VWF A1‐platelet GPIbα interaction dynamics,^58^, ^59^ however the potential of these peptides for designing hemostatic nanotechnologies is yet to be evaluated. In our research, for vWF‐binding peptide (VBP) we have utilized the sequence TRYLRIHPQSWVHQI derived from the C2 domain (residues 2303‐2332) of the coagulation factor FVIII that binds to vWF D′‐D3 domain. For collagen‐binding peptide (CBP), we have utilized a 7‐mer repeat of the Glycine(G)‐Proline(P)‐Hydroxyproline(O) tri‐peptide (i.e. [GPO]_7_) that has helicogenic affinity to fibrillar collagen but minimal ability to activate platelets via GPVI (hence minimal systemic thrombotic risk). We demonstrated that VBP‐decorated liposomes can undergo shear‐dependent adhesion onto vWF‐coated surfaces or on collagen surfaces in presence of soluble vWF.^60^ The fact that VBP binds to vWF D′‐D3 domain and not the GPIbα‐interactive A1 domain, allows the VBP‐decorated liposomes to bind vWF without competing with endogenous platelet adhesion to the same vWF. CBP‐decorated liposomes exhibited significant binding to collagen‐coated surfaces under flow, at all shear ranges. Inspired by the synergistic ‘vWF + collagen’ adhesion of platelets, we have also investigated the co‐decoration of VBP and CBP on liposomes, and the resultant particles showed significantly higher localization on ‘vWF + collagen’‐coated surfaces at low‐to‐high shear ranges, compared to liposomes bearing VBP only or CBP only.^61^

### 3.4 Hemostatic nanomedicine combining multiple platelet mechanisms

**FIGURE 2:**
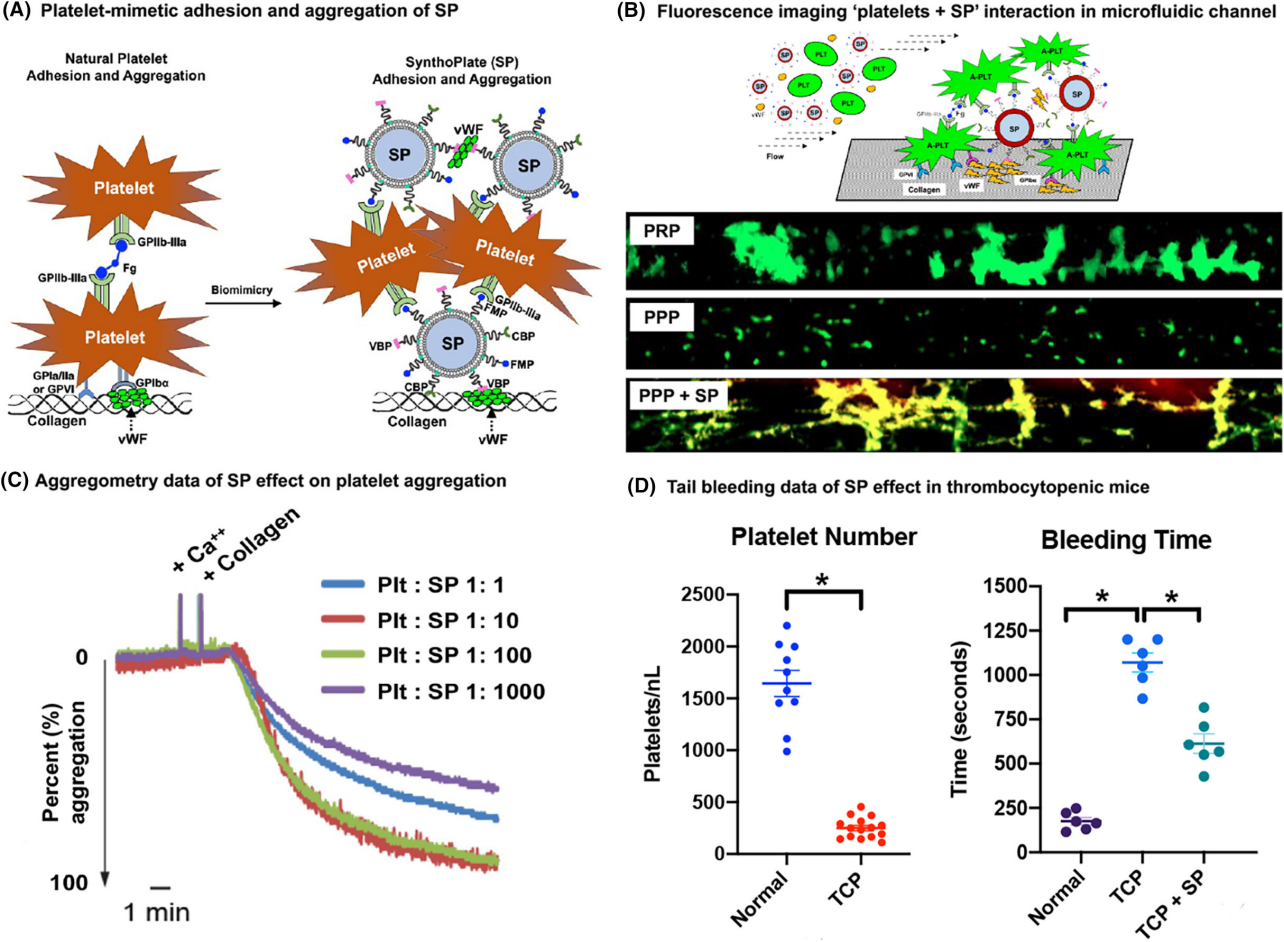
(A) SynthoPlate (SP) mimics platelet adhesion and aggregation mechanisms by binding to vWF vis vWF‐binding peptide (VBP), to collagen via collagen‐binding peptide (CBP), and to active platelet GPIIb‐IIIa via fibrinogen‐mimetic peptide (FMP); (B) Microfluidic set‐up and representative fluorescence microscopy images, where calcein‐stained (green) platelets and soluble vWF in plasma is flowed over collagen‐coated channel at high shear (60 dyn cm^−2^): PRP was able to form large platelet aggregates on the channel surface, and this was significantly reduced with PPP; introducing Rhodamine B labeled (red) SP in PPP significantly rescued platelet aggregate formation (red SP colocalization with green platelets shown in yellow); (C) shows representative lumi‐aggregometry results of SP effect on platelet aggregation where Platelet (Plt): SP ratio of 1:10 to 1:100 increased platelet aggregation, below this ratio (Plt: SP = 1:1) had negligible effect, while above this ratio (Plt: SP = 1:1000) reduced aggregation possibly due to dilution effect; (D) Representative results in mouse tail‐clip bleeding model shows that induction of thrombocytopenia (TCP) in mice significantly increased bleeding time, and treatment of this TCP condition with SP reduced bleeding time significantly

Building on the above approaches, we have investigated combining both *aggregation* and *adhesion* mechanisms on a single particle platform. Here we have used the terminology ‘heteromultivalent modification’ (*hetero*: different types, *multi*: many, *valency*: interactivity) to reflect the pluraility of simultaneous heterotypic interactions. In fact, the designs described previously that involve particle surface‐decoration with combination of ‘rGPIbα + rGPIa‐IIa’ or ‘VBP + CBP’ to simulate ‘vWF + collagen’ binding, also fall in this *heteromultivalent* category. To combine platelet ‘adhesion + aggregation’ on a single particle, previous approaches including ours have explored surface‐decoration with a combination of ‘rGPIbα + H‐12 peptides’ and ‘rGPIbα + FMP peptides’. The hemostatically relevant outputs of these designs were compared *in vitro* to rGPIbα‐decorated particles only, H‐12 or FMP‐decorated particles only, and a physical mixture of ‘rGPIbα‐decorated + H‐12 (or FMP)‐decorated particles’.^61^, ^62^ These studies have indicated that the significant size difference between the large rGPIbα fragment compared to small H‐12 or FMP peptide can reduce the synergistic functional output, due to steric masking of the smaller motif by the larger one. Therefore, we shifted to using particle surface‐decorations with small peptide combinations only. To this end, we have created a liposome‐templated design that is surface‐decorated with a combination of VBP, CBP and FMP peptides to mimic ‘adhesion + aggregation’ mechanisms of platelets and have named this *synthetic platelet* design SynthoPlate.^63^, ^64^, ^65^, ^66^ Our *in vitro* and *in vivo* studies with SynthoPlate have demonstrated that this functional integration leads to higher hemostatic efficacy compared to particles bearing adhesion functionality only or aggregation functionality only. This technology has demonstrated promising hemostatic efficacy in mouse thrombocytopenia model, mouse and rat acute liver injury model and pig femoral artery hemorrhage model. SynthoPlate can be effectively sterilized and stored as aqueous suspension for up to 9 months without affecting platelet‐mimetic bioactivity, and thus can potentially serve as a platelet surrogate when donor platelets are unavailable. Figure 2 shows some representative results of SynthoPlate effect on enhancing platelet recruitment and aggregation to improve hemostasis in a thrombocytopenic setting. Current translational development of SynthoPlate is being conducted by Haima Therapeutics, regarding advancing the technology as an aqueous‐reconstitutable lyophilized powder, for on‐demand intravenous hemostatic use in hospital and field settings.

### 3.5 Emergent designs in platelet‐inspired hemostatic technologies

**FIGURE 3:**
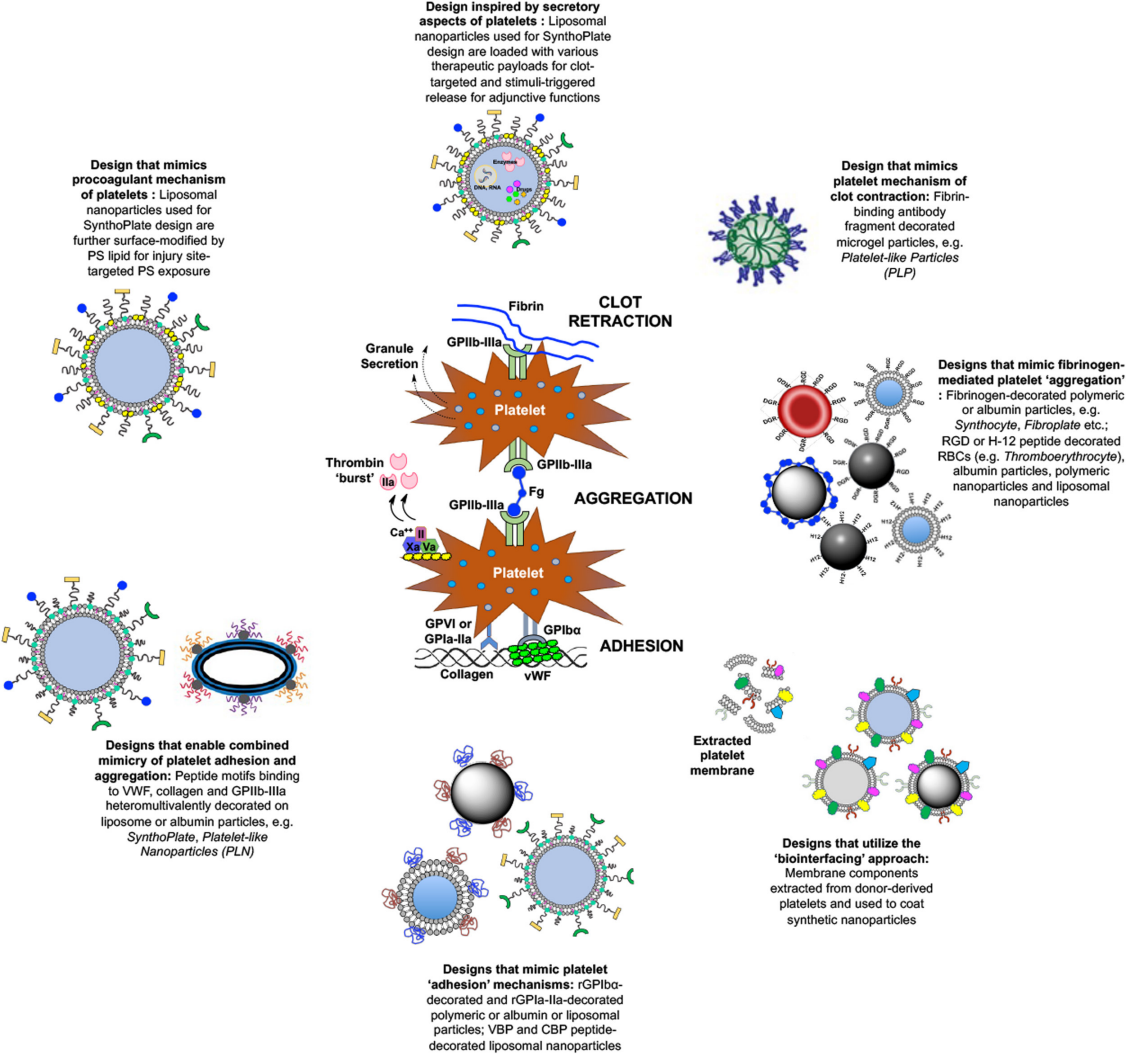
Various design approaches for platelet‐inspired hemostatic nanomedicine constructs

Figure 3 depicts the various design approaches for platelet‐inspired hemostatic technologies, that were described in the previous sections. These approaches have also led to exploring additional platelet‐inspired design components to augment the hemostatic performance. One exciting approach is the exploration of morphological characteristics of platelets that influence their hemostatic responses. Circulating *resting* platelets have biconvex discoid shape with 2–5 μm diameter, 0.5 μm thickness and an elastic modulus of 10–50 kPa.^67^ In comparison, circulating healthy RBCs are biconcave discoid in shape, with approximately 8 μm diameter and much lower elastic modulus (≦10 kPa). Mathematical modeling and experimental analyses have indicated that these key *biophysical* differences between RBCs and platelets lead to the expulsion of platelets from the RBC bulk flow volume and their margination closer to the blood vessel wall.^68^ This margination enhances platelet's collision probability with the wall and in turn augments their rapid hemostatic responses.^69^ Based on this, several research groups including ours have investigated the incorporation of platelet‐mimetic geometry in ligand‐decorated particle design, to integrate biophysical and biochemical parameters. These studies indicated that particles that are of platelet shape (oblate or discoid) and size (~2 μm diameter) have improved interactive capability on target surfaces in presence of hematocrit, compared to spherical nanoscale particles.^70^, ^71^ The current translational barrier to this approach is the limited scale at which such anisotropic particles can be manufactured. However, with advanced manufacturing techniques emerging, one can envision that future platelet‐inspired particle technologies can overcome this barrier. Furthermore, future studies can explore unique particles systems that undergo stimuli‐responsive dynamic shape changes analogous to the morphological transformations of *resting* platelets to *activated* platelets.

Another interesting strategy is the utilization of platelet‐inspired particles as carrier platforms for adjunctive hemostatic agents. As stated earlier, the H12‐peptide decorated liposomes have been studied for the delivery of ADP (a platelet agonist).^49^ In analogous approach, we have studied the loading of tranexamic acid (TXA, a plasmin inhibitor) using FMP‐decorated liposomes for clot‐targeted delivery to treat trauma‐associated hyperfibrinolysis.^72^ We have also recently investigated the potential of directly delivering thrombin using ‘VBP + CBP’‐decorated liposomes for injury‐targeted generation of fibrin in treating coagulopathic bleeding.^73^ Due to the important role of platelet‐derived PolyP in modulating coagulation kinetics and clot structure, some approaches are also exploring PolyP delivery using nanoparticle platforms.^74^, ^75^ In another recent approach, we have explored the exposure of anionic phospholipids (e.g. phosphatidylserine, PS) on the surface of SynthoPlate nanoparticles in an injury site‐selective manner, inspired by the platelet procoagulant function.^76^ Here, the PS remained masked by a polyethylene glycol (PEG) brush conjugated on the particle surface, which could be cleaved by the action of plasmin predominantly at the injury site for targeted augmentation of hemostasis. This new design could significantly enhance hemostasis, even when endogenous platelet activity was impaired. Some approaches are also focusing on synthetic biology tools to attempt the mimicry of more complex platelet signaling mechanisms and protein expression in phospholipid vesicles.^77^ Altogether, the research in platelet‐inspired hemostatic nanotechnologies continues to provide a variety of customized therapeutic opportunities to treat various bleeding complications. Recent research has also emphasized the promise of incorporating such *platelet surrogates* with other blood components to potentially create *biosynthetic whole blood* systems for transfusion applications.^78^, ^79^

## 4 NANOMEDICINE INSPIRED BY PLATELET ROLE IN THROMBOSIS AND THROMBOINFLAMMATION

### 4.1 Platelets in thrombosis and relevant therapeutic strategies

**FIGURE 4:**
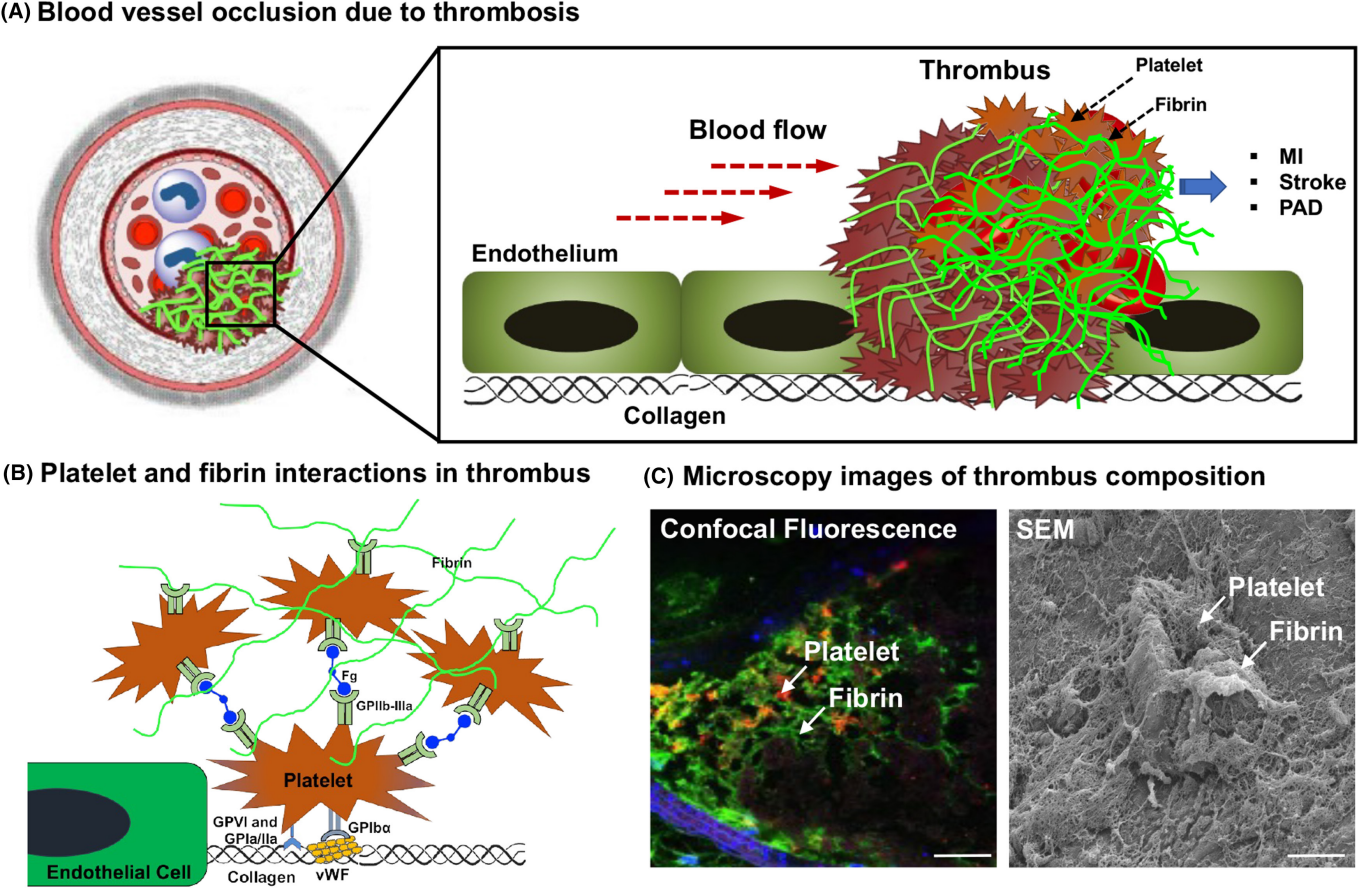
Platelet involvement in thrombosis: (A) shows a schematic of how vaso‐occlusive thrombus can obstruct blood flow and lead to pathologic conditions like myocardial infarction (MI), stroke and peripheral arterial disease (PAD); (B) shows specific interactions in thrombi where activated platelets can adhere to vWF (via GPIbα) and exposed collagen (via GPIa/IIa and GPVI), and aggregate via binding of platelet GPIIb‐IIIa to fibrinogen (Fg) and fibrin; (C) shows representative confocal fluorescence and scanning electron microscopy (SEM) images of ‘platelets + fibrin’ ‐rich occlusive thrombus in mouse arterial thrombosis model

The cellular and molecular mechanisms of hemostasis, when dysregulated, lead to the formation of occlusive blood clots, termed thrombosis. In healthy blood vessels, the luminal wall is lined by endothelial cells (ECs) sitting on a subendothelial matrix of collagen.^80^ These healthy ECs present a dense brush of carbohydrate‐rich polymers on their blood‐contacting surface, termed the *glycocalyx*, that renders thromboresistance via multiple steric, antiplatelet and anticoagulation mechanisms.^81^ Vascular pathologies that injure and denude this endothelium result in thrombus formation. Anatomically, thrombosis can be arterial or venous, and platelets are significantly involved in both.^82^ Platelet involvement in arterial thrombosis (Figure 4) stems from its ability to undergo *adhesion* to exposed vWF and collagen at the site of endothelial damage, *activation* by multiple autocrine and paracrine agonists (e.g. collagen, ADP, TXA_2_, thrombin etc.), *aggregation* via activated platelet GPIIb‐IIIa binding to fibrinogen, fibrin, VWF and fibronectin, and procoagulant *thrombin amplification* for enhanced fibrin generation.^3^, ^83^, ^84^, ^85^

Therefore, a significant number of therapeutic approaches focus on inhibiting these mechanisms, by pharmacological inhibition of platelet *activation*, platelet *adhesion* and platelet *aggregation*.^86^ In parallel to these *anti‐platelet* agents, several drugs focus on *anti‐coagulant* effect by inhibition of coagulation factors, Vitamin K, thrombin etc. While such agents work by preventing or reducing thrombus growth, on the other end of the spectrum are *thrombolytic* agents (e.g. tissue plasminogen activator, tPA) that work by breaking down fibrin.^87^, ^88^ Therefore, the current pharmacological approaches for treating thrombosis rely heavily on reducing clot‐making and enhancing clot‐breaking mechanisms. However, all of these approaches are currently administered systemically (oral or intravenous), and this persistently presents a bleeding risk since the drugs affect the body's natural hemostatic status. This is where platelet‐inspired nanomedicine approaches may provide unique *disease‐targeted* strategy, to enhance therapeutic efficacy while avoiding systemic and off‐target side‐effects.

### 4.2 Platelets in thromboinflammation and relevant therapeutic strategies

**FIGURE 5:**
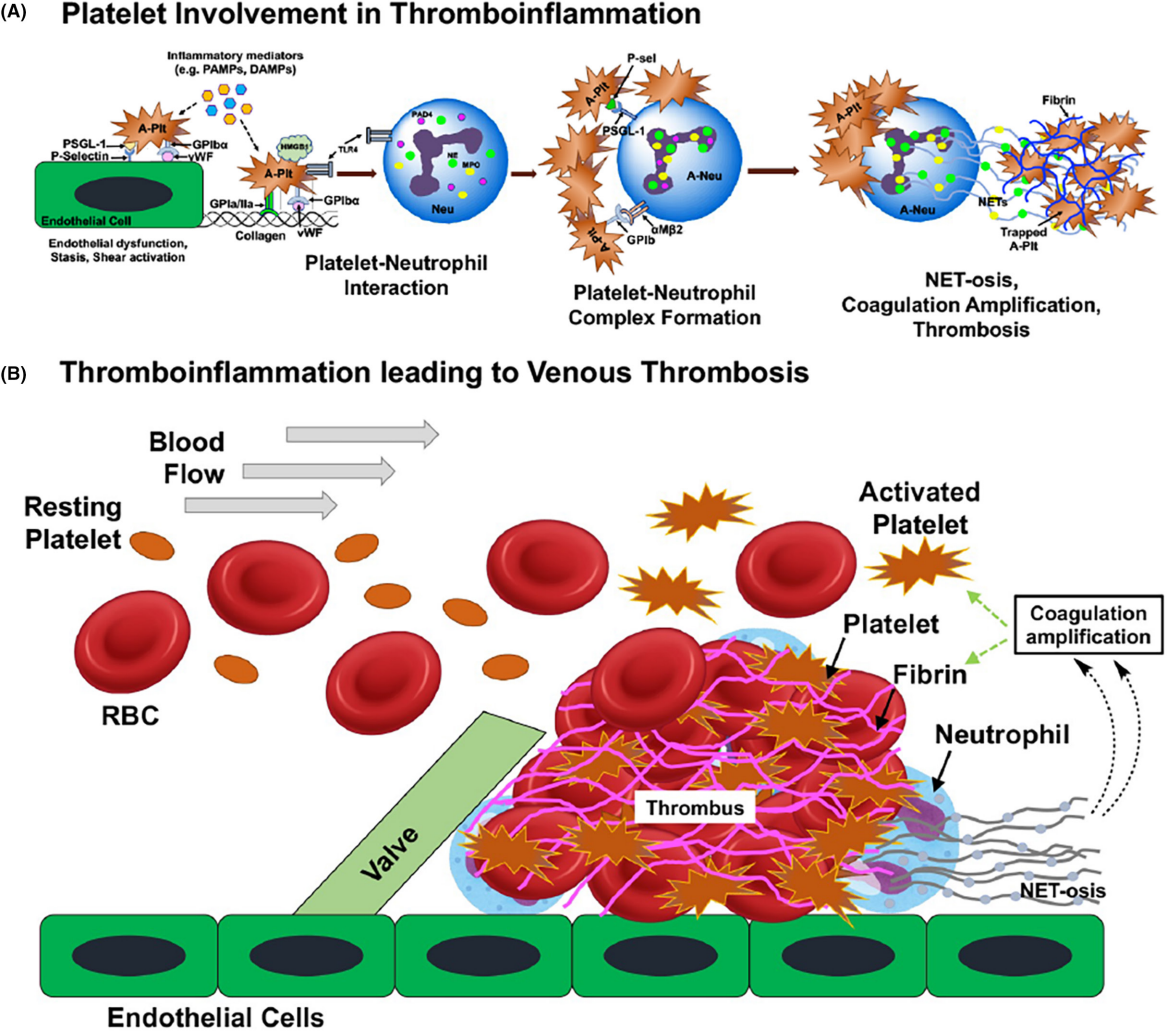
Platelet involvement in thromboinflammation and venous thrombosis: (A) shows representative heterotypic interactions between platelets and neutrophils leading to neutrophil extracellular trap formation (NET‐osis); (B) shows further complexation of such platelet‐neutrophil interactions and NET‐osis leading to coagulation amplification, fibrin formation and platelet aggregation in venous thrombus growth

Platelets are also a major driver of thromboinflammation, a complex pathology that involves heterotypic interaction of platelets with immune and endothelial cells. Platelet activation is associated with the upregulation of immunomodulatory molecules like P‐selectin on their surface. The binding between platelet P‐selectin with P‐selectin Glycoprotein Ligand‐1 (PSGL‐1) on immune cells initiates platelet‐leukocyte interactions, which is further stabilized by the direct interaction of platelet GPIbα with the macrophage‐1 antigen (MAC‐1, α_M_ß_2_), and fibrinogen‐mediated interaction of platelet GPIIb/IIIa with MAC‐1. These heterotypic interactions (Figure 5A) are a hallmark of thromboinflammatory pathologies in deep vein thrombosis (DVT), pulmonary microvascular occlusion, sepsis etc.^6^, ^89^, ^90^, ^91^, ^92^, ^93^, ^94^. Activated platelets also release CD40L from α‐granules, which interacts with CD40 on leukocytes increasing their recruitment and activation. Furthermore, platelet CD40L can upregulate tissue factor, E‐selectin, VCAM‐1 and ICAM‐1 on endothelial cells supporting a procoagulant phenotype. Platelet‐neutrophil interactions lead to neutrophil activation, secretion of elastase, myeloperoxidase, S100 A8/A9, histones etc., and extrusion of DNA as neutrophil extracellular traps (NETosis). NETosis is an obligate innate immune response of neutrophils to neutralize pathogens, but aberrant NETosis result in pathologic thrombosis both in sterile and infectious diseases. Platelet activation directly supports NETosis via the expression of P‐selectin and high mobility group box 1 (HMGB‐1) protein, and NETs contribute to procoagulant mechanisms of thrombus growth (Figure 5B). Due to its key role in initiating platelet‐leukocyte interactions, P‐selectin has emerged as a therapeutic target to reduce thrombosis in many thrombo‐inflammatory diseases including DVT^95^ and sickle cell disease. In a recent therapeutic development, Crizanlizumab, a humanized monoclonal antibody to P‐selectin, was clinically approved as a treatment for limiting thromboinflammation in sickle cell disease associated vaso‐occlusive crisis.^96^ Inhibitors that block the interaction of platelet GPIbα with MAC‐1 can also reduce thrombosis.^92^ Other platelet‐associated receptor‐ligand interactions such as programmed cell death protein 1 (PD)‐1‐PD ligand‐1 (PDL‐1) can also exert immunomodulatory functions in thromboinflammation.^97^ Recently, platelet ITAM receptors C‐type lectin‐like receptor *2* (CLEC‐2) and GPVI have emerged as novel targets in thromboinflammatory diseases, due to the finding that CLEC‐2 interaction with its ligand podoplanin promotes venous thrombosis.^98^, ^99^ Beside its prothrombotic role, CLEC‐2‐podoplanin interaction has also been implicated in acute respiratory distress syndrome (ARDS), sepsis and peritoinitis in mice.^100^, ^101^, ^102^ Such findings suggest the therapeutic potential of targeting CLEC‐2 and GPVI interactions to regulate thrombosis and thromboinflammation. Emerging research during the current COVID‐19 pandemic has revealed that COVID‐19 patients have hyperactive and procoagulant platelets, as well as platelet‐leukocyte aggregates characteristic of thromboinflammation.^103^, ^104^, ^105^ Platelets and plasma of COVID‐19 patients were found to contain elevated levels of S100A8/A9 and HMGB1, that can cause endotheliopathy and thromboinflammation.^106^ The above findings across various pathologies present unique opportunities for platelet‐inspired nanomedicine platforms as an innovative strategy for disease site‐targeted therapies. Potetial payload for such platforms can be anti‐platelet and anti‐coagulant agents, neutrophil function modulating agents, NET‐degrading and fibrinolytic enzymes, etc.

### 4.3 Platelet‐inspired nanomedicine approaches for thrombosis and thromboinflammation

Several approaches have been investigated leveraging the involvement of platelets in thrombosis and thromboinflammation for targeted drug delivery. One such approach is the direct chemical modification of drugs to enable binding to thrombus‐associated activated platelets. For example, urokinase was modified with a monoclonal antibody 7E3 (Abciximab) that binds to platelet integrin GPIIb‐IIIa.^107^ This urokinase‐7E3 system demonstrated targeted fibrinolytic and antiplatelet ability ex vivo at lower concentrations compared to free urokinase. An analogous design involved an engineered staphylokinase (SAK) mutant bearing platelet GPIIb‐IIIa‐binding RGD sequence.^108^ This SAK‐RGD system showed enhanced platelet targeting and fibrinolytic ability *in vitro* and efficient clot lysis *in vivo* in pigs. In yet another approach, single‐chain urokinase plasminogen activator (scUPA) was conjugated to an antibody fragment (scFv) specific for platelet GPIIb‐IIIa, to enable targeting to thrombi in mice, without affecting hemostasis.^109^ In yet another strategy, platelet GPIIb‐IIIa‐targeting scFv was conjugated to recombinant microplasminogen activable by thrombin, such that thrombin‐triggered release of platelet‐targeted microplasminogen could enable local clot lysis in mouse model.^110^ In an interesting design to enable ‘clot‐targeted triggerable release of tPA’, albumin was conjugated to tPA via a thrombin‐cleavable peptide sequence GFPRGFPAGGC and then the albumin shell was decorated with platelet GPIIb‐IIIa targeting CQQHHLGGAKQAGDV peptide.^111^ This construct was able to bind to activated platelets *in vitro* and target clots *in vivo* to render fibrinolytic activity at levels equivalent to free tPA, but with reduced systemic side‐effects.

In contrast to directly modifying a drug with ligands, several approaches have focused on packaging the drug within clot‐targeted nanoparticles. Packaging of fibrinolytic drugs like streptokinase (SK) and tPA within nanoparticles was first attempted to improve drug circulation time.^112^, ^113^, ^114^ For example, liposome‐encapsulation of tPA increased its circulation lifetime by 4–5 fold compared to free tPA, and once released, the tPA could render effective fibrinolysis. Another particle system used for such studies is ultrasound‐sensitive bubbles made of perfluorocarbon (PFC) encapsulated within a lipidic or polymeric shell. Such bubbles not only act as a carrier for drugs, but via ultrasound‐mediated image guidance and bubble cavitation they can enable site‐localized drug release. This ultrasound‐triggerable approach has led to the concept of ‘sonothrombolysis’.^115^, ^116^ Building on such approaches, researchers have also investigated the surface‐decoration of such particles and bubbles with clot‐targeted anchoring motifs. We and others have utilized the platelet GPIIb‐IIIa‐targeting RGD ligands to decorate nanoparticles loaded with thrombolytic drugs, and this enabled targeted action of the drug *in vitro* and in murine models *in vivo*.^117^, ^118^, ^119^, ^120^ In further advancement of this approach, our work has focused on co‐decorating drug‐loaded liposomes with a combination of active platelet GPIIb‐IIIa‐binding and P‐selectin‐binding peptides, to enhance the clot‐targeting capability and therapeutic effect of the particles.^121^ In yet another heteromultivalent approach, liposomes were surface‐decorated with a combination of GPIIb‐IIIa‐binding and fibrin‐binding peptides, and this design maximized the clot‐localization of the nanoparticles under shear flow.^122^ The Poly‐NIPAM based low‐crosslinked fibrin‐binding gel particles stated previously (see Section 2) have also recently been shown to deliver tPA for fibrin‐targeted treatment of disseminated intravascular coagulation.^123^

**FIGURE 6:**
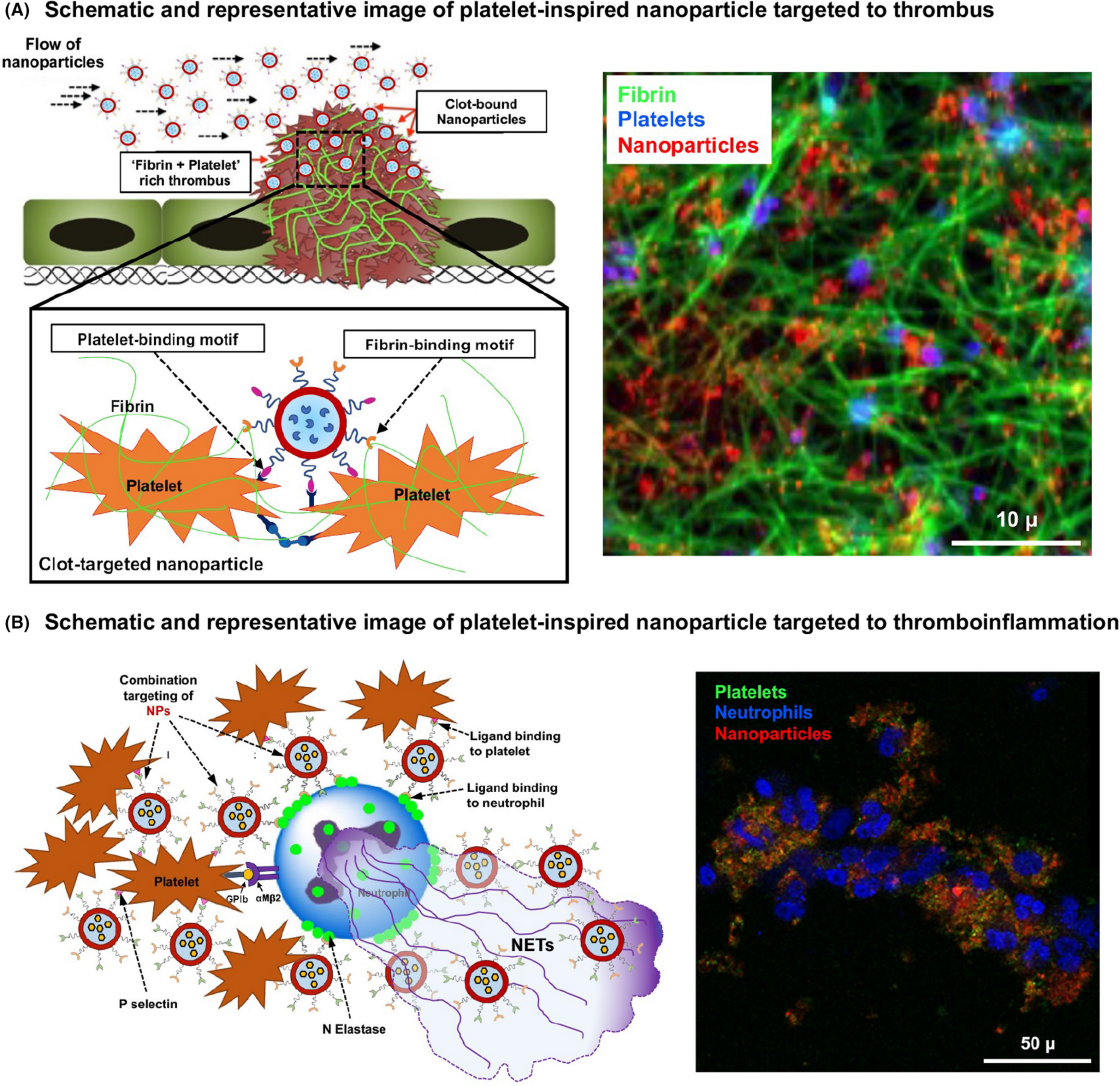
Design schematic and representative fluorescence images of platelet‐inspired nanoparticles targeted to (A) thrombotic (e.g. binding platelets and fibrin) and (B) thromboinflammatory (e.g. binding platelets and neutrophils) pathologies

In thromboinflammatory pathologies, current treatments predoiminantly use systemic (e.g. oral or intravenous) administration of drugs, which can pose harmful bleeding side effects. Here, nanomedicine approaches that can specifically target platelets or platelet‐leukocyte or platelet‐endothelium complexes, can provide unique avenues for site‐specific therapy with enhanced systemic safety. For example, we have recently developed liposomal nanoparticles capable of molecular anchorage to activated platelet‐neutrophil complexes, via particle surface‐decoration with P‐selectin binding peptides (PBP) and neutrophil elastase binding peptides (NEBP), and the resultant constructs were able to bind DVT‐relevant thrombi *in vitro* and in murine models *in vivo*.^124^ In another example, lipid‐polymer hybrid nanoparticles was surface‐decorated with a peptide sequence KZWXLPX (Z: hydrophobic amino acid, X: any amino acid) to actively target collagen IV at arterial injury sites and deliver anti‐proliferative agents for modulating smooth muscle cell activity.^125^ In a similar approach, micellar nanoparticles were surface decorated with a 9‐amino acid sequence CGNKRTRGC that binds to p32 receptors in atherosclerotic plaques, as well as, with CREKA peptides that bind to fibrin‐fibronectin clots, and these micelles showed enhanced targeting ability to atherosclerotic plaques *in vivo*.^126^ One can envision utilizing such platforms for targeted drug delivery across various thromboinflammatory pathologies. Figure 6 shows specific examples from our own research on platelet‐inspired nanomedicine systems targeted to thrombotic and thromboinflammatory niche, along with example fluorescence images of nanoparticle binding.

**FIGURE 7:**
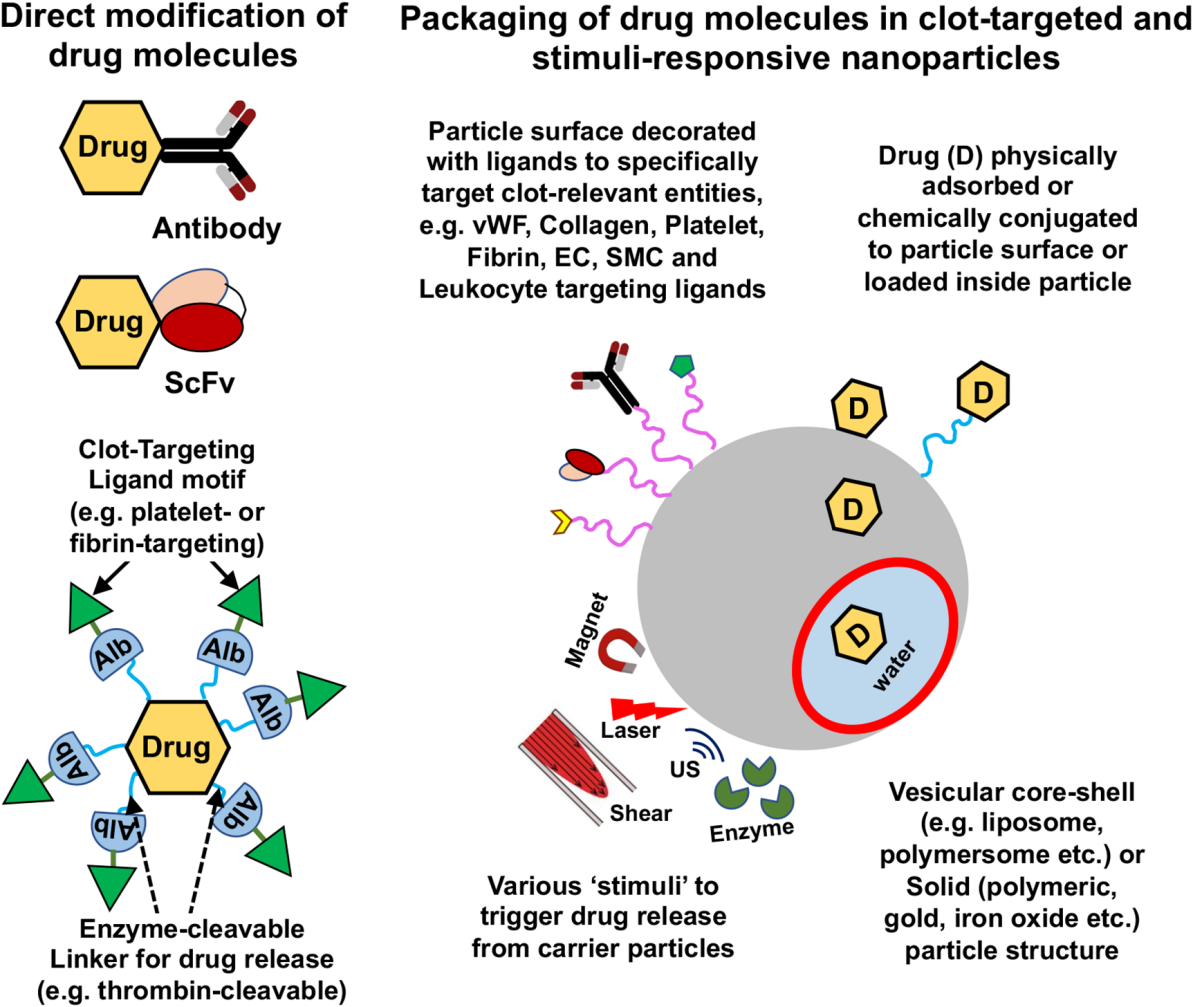
Platelet‐inspired nanomedicine approaches in thrombosis and thromboinflammation: Therapeutic agents can be modified directly with targeting ligands (e.g. antibodies, antibody fragments, peptides etc.) that bind to clot‐specific entities (e.g. activated platelets, fibrin, leukocytes etc.), or they can be packaged with nanoparticles surface‐decorated with such ligand motifs; Release of surface‐immobilized or encapsulated drug from the particles can be triggered by various endogenous (e.g. enzyme, shear) or external (e.g. magnetic, laser, ultrasound) stimuli

While delivering the drug in a site‐specific manner is one aspect of such nanoparticle designs, another important design requirement is the release of the drug payload once the particles are localized at the target site. In majority of research reported so far, the drug release is rendered by diffusion. However, in recent years several unique particle designs have been reported that utilize endogenous (e.g. enzymes, shear) or externally applied (e.g. magnetic, thermal, ultrasound, etc.) stimuli to trigger site‐specific drug release.^127^, ^128^ These stmuli‐triggered release mechanisms can be potentially combined with the platelet‐inspired clot‐targeted delivery strategies, to create unique therapeutic technologies directed at thrombotic and thromboinflammatory pathologies. Figure 7 shows schematic of nanomedicine approaches where platelet‐targeted (and other clot component‐targeted) ligands can be conjugated directly to the drug, or the drug can be packaged within nanoparticles surface‐decorated with such ligands, for targeted delivery and stimuli‐triggerd release at the clot site for enhanced treatment efficacy with minimal systemic effects.

## 5 DISCUSSION

The multifunctional roles of platelets in hemostasis thrombosis and thromboinflammation provide unique design cues for the engineering of nanomedicine strategies specifically targeted to these conditions. The fundamental design approach for such strategies is to elucidate specific cellular and molecular mechanisms in such pathologic microenvironment, and then mimic or leverage these mechanisms on appropriate ligand‐decorated nanoparticles that encapsulate specific drug molecules. To this end, particle systems utilizing platelet‐inspired design approaches have shown encouraging results in preclinical *in vitro* and *in vivo* models. Their clinical translation will require rigorous evaluation of their manufacturing and scale‐up, demonstration of batch‐to‐batch reproducibility regarding physico‐chemical and biointeractive properties, and appropriate evaluation of their pharmacological and toxicological profile. During the last two decades there has been a significant advancement of nanmedicine systems towards clinical trials and approvals, with the latest example being the delivery of COVID‐19 mRNA vaccines using a lipid nanoparticle platform.^129^ Therefore, one can envision exciting therapeutic endeavors in the cardiovascular area using platelet‐inspired nanomedicine platforms in the near future.

## CONFLICT OF INTEREST

A.S.G. is an inventor on patents US 9107845B2, US 9636383B2, US 10426820B2, US 10434149B2, on ‘Synthetic Platelet’ technologies. A.S.G. is also a co‐founder of Haima Therapeutics where these patents are licensed. A.S.G. is also an inventor on patent US 9107963 for platelet‐inspired drug delivery platform. S.R. and J.R. have nothing further to disclose.

## AUTHOR CONTRIBUTIONS

S.R. contributed to writing sections on platelet role in hemostasis, thrombosis and thromboinflammation, and some sections on platelet‐inspired nanomedicine technologies. J.R. contributed to writing sections on platelet role in thromboinflammation and platelet mechanisms in COVID‐19. A.S.G. wrote sections on platelet mechanisms in hemostasis and thrombosis, as well as sections on platelet‐inspired nanomedicine technologies, prepared all schematic figures, and compiled the manuscript.
